# Early Detection of Depression: Social Network Analysis and Random Forest Techniques

**DOI:** 10.2196/12554

**Published:** 2019-06-10

**Authors:** Fidel Cacheda, Diego Fernandez, Francisco J Novoa, Victor Carneiro

**Affiliations:** 1 Department of Computer Science Faculty of Computer Science University of A Coruna A Coruna Spain; 2 Center for Information and Communications Technology Research University of A Coruna A Coruna Spain

**Keywords:** depression, major depressive disorder, social media, artificial intelligence, machine learning

## Abstract

**Background:**

Major depressive disorder (MDD) or depression is among the most prevalent psychiatric disorders, affecting more than 300 million people globally. Early detection is critical for rapid intervention, which can potentially reduce the escalation of the disorder.

**Objective:**

This study used data from social media networks to explore various methods of early detection of MDDs based on machine learning. We performed a thorough analysis of the dataset to characterize the subjects’ behavior based on different aspects of their writings: textual spreading, time gap, and time span.

**Methods:**

We proposed 2 different approaches based on machine learning singleton and dual. The former uses 1 random forest (RF) classifier with 2 threshold functions, whereas the latter uses 2 independent RF classifiers, one to detect depressed subjects and another to identify nondepressed individuals. In both cases, features are defined from textual, semantic, and writing similarities.

**Results:**

The evaluation follows a time-aware approach that rewards early detections and penalizes late detections. The results show how a dual model performs significantly better than the singleton model and is able to improve current state-of-the-art detection models by more than 10%.

**Conclusions:**

Given the results, we consider that this study can help in the development of new solutions to deal with the early detection of depression on social networks.

## Introduction

### Background

Major depressive disorder (MDD), also known simply as depression, is among the most prevalent psychiatric disorders globally [1,2]. As described in the World Health Organization’s Comprehensive Mental Health Action Plan 2013-2020 [3], depression alone affects more than 300 million people worldwide and is one of the largest single causes of disability worldwide, particularly for women. Depression currently accounts for 4.3% of the global burden of disease, and it is expected to be the leading cause of disease burden in high-income countries by 2030 [4].

The Institute of Medicine Committee on the Prevention of Mental Disorders identified depression as the most preventable disorder [5], and several studies have demonstrated that early recognition and treatment of depression can improve the negative impacts of the disorder [6-8]. Therefore, it is vital to provide an early identification of subjects suffering from depression to intervene as soon as possible and minimize the impact on public health by potentially reducing the escalation of the disease. However, provisions and services for the early detection and treatment of depression and other mental health disorders remain limited. Although there are also some validated laboratory tests to diagnose depression, such as Beck Depression Inventory‐II, Center for Epidemiologic Studies Depression Scale (CES‐D), Geriatric Depression Scale, Hospital Anxiety and Depression Scale, Patient Health Questionnaire‐9 [9,10], and Hamilton Rating Scale for Depression [11] most diagnoses are formed on the basis of self- or family reports.

In this context, the relation between language and clinical disorders has been analyzed for years [12,13]. Taking this into account, new work has appeared to predict and study depression [14,15]. In particular, researchers are increasingly examining the potential of social media networks as tools to predict depression and detect its symptoms as manifested in user comments and related activities. Social networks such as Twitter, Inc, Facebook, Inc, and Reddit, Inc have become part of our daily lives as media through which to share our thoughts, feelings, and overall emotional status. As such, these platforms have become valuable data banks for marketers and researchers, who can analyze user metrics, shared content, and related information to identify preferences and tastes as well as other attitudes and behaviors [15,16]. In fact, social networks have proved to be used by patients to interact with peers because of their support and ability to understand someone’s experience, while maintaining a comfortable emotional distance [17]. For example, Reddit, Inc is an open-source platform where community members can submit content and vote on submissions. Content entries are organized by areas of interest (denoted as subreddits), with a large history of previous submissions covering several years. This social network is particularly interesting for our study, as it contains substantive content about different medical conditions, including MDD.

This study uses publicly available data from Reddit, Inc to examine the effectiveness of different methods that can provide an early detection of MDDs based on artificial intelligence. As detailed in the next sections, we mainly focus on 2 different methods, both of which are based on machine learning algorithms that use textual and semantic similarity features along with writing features (WFs) to predict a subject’s depression condition. The first technique follows a simpler proposal using a single machine learning algorithm, whereas the second model follows a dual approach that uses 2 machine learning algorithms: the first one is trained to predict depression cases, whereas the second one is trained to predict nondepression cases. We conducted a thorough evaluation of each model following a time-aware approach that rewards early detections and considers late detections as false negatives. Our results show that the dual model can improve state-of-the-art detection models up to 10%. Furthermore, our methods were implemented using freely available tools, thus facilitating the reproduction of our research work [18].

The aim of this study was to explore the use of machine learning for an early detection of MDD using WFs from social network content to improve state-of-the-art methods, which can lead to the development of early detection technologies that could help in the identification of subjects suffering from depression. The main contributions of our study can be summarized as follows:

- We provide a detailed analysis on publicly available data from social networks to characterize the subjects’ behavior based on different aspects of their writings: textual spreading, time gap, and time span.
- We propose 2 different machine learning methods, named singleton and dual, that use textual, semantic, and WFs derived from subjects’ social networks behavior to predict his depression condition.
- We follow a time-aware evaluation that strictly penalizes late depression detections. Our results show that the dual model is able to improve upon state-of-the-art methods.

The structure of the paper is as follows. First, we examine related studies with regard to early detection of depression with a particular focus on techniques that use information extracted from social networks. Then, we provide a detailed data analysis of the social network content for MDD detection and we describe our proposed model for the early detection of depression. After the methods, we present the results and performance improvements obtained over the state-of-the-art baselines. Finally, we summarize our conclusions and future studies in this line of research.

### Related Studies

Several previous studies have highlighted the importance of early detection in improving outcomes related to MDD [6-8]. Halfin’s study [6] demonstrated that the early detection, intervention, and appropriate treatment can promote remission and reduce the emotional and financial burdens of this disease, and Picardi et al [7] observed significant improvements in depressive symptoms and quality of life among subjects who had undergone early screening. Rost et al [8] found that early intervention for depression can improve employee productivity and reduce absenteeism.

Over the past decade, social networks have increasingly become a focus of research efforts to identify and characterize the incidence of various disorders. For example, Prieto et al [19] proposed a method to use Twitter, Inc to automatically measure the incidence of a set of health conditions. Chunara et al [20] analyzed cholera-related tweets published during the first 100 days of the 2010 Haitian cholera outbreak, and Chew and Eysenbach [21] used sentiment analysis on 2 million tweets to propose a complementary infoveillance approach. Aladağ et al [22] have studied posts looking for regular language patterns to prevent potential suicide attempts. Even Rice et al [23] have demonstrated that the development of cost-effective, acceptable, and population-focused interventions is critical in depression. A number of online interventions (both prevention and acute phase) have been tested in young people with promising results.

Diverse studies have explored the potential of social media networks to predict and detect mental health disorders [24-28]. For example, De Choudhury et al [27] developed a statistical methodology to derive distinct markers of shifts to suicidal ideation from Reddit, Inc user data for modeling in a prediction framework, and Birnbaum et al [25] proposed a method that used machine learning in combination with clinical appraisals as a means of identifying social media markers of schizophrenia.

Other studies have focused specifically on depression. Ziemer and Korkmaz’s [29] comparison of human versus automated text analyses of psychological and physical disorders found human ratings of depression to be more accurate than machine-based methods; however, other studies have yielded promising, albeit limited, results using sophisticated technological applications in detecting and measuring the disorder. Nadeem’s *bag of words* analysis of Twitter, Inc messages [30] examined the frequency of use of *my* and *me* as a marker for depression, whereas De Choudhury et al [15] leveraged social activity, emotion, and language signals manifested on Twitter, Inc to introduce a social media depression index. Similarly, a task organized at the Computational Linguistics and Clinical Psychology Workshop 2015 to detect depression and other mental health disorders among subjects using Twitter, Inc posts achieved promising results using topic modeling and rule-based methods [31-33].

Fewer studies have focused on early detection of depression. Ophir et al [34] examined signals of depression among adolescent Facebook, Inc users with the aim of ultimately applying their coding scheme to early detection methods, although no methods are proposed by the authors. De Choudhury et al [15] achieved 70% accuracy in an experiment that compared scores found on the Center for Epidemiologic Studies Depression Scale [35] and BDI [36] with Twitter, Inc users’ engagement patterns and linguistic markers preceding a recent episode of depression to devise a tool for predicting and measuring MDD in individuals. This study identified several distinctive features of posting activity associated with the onset of depression, such as diurnal cycles, more negative emotions, less social interaction, more self-focus, and more mentions of depression-related terms. However, as with most other research that attempts to predict depression, the analysis was dependent on self-reported cases, and to date, approaches aiming to identify individuals who are as yet unaware of their depression diagnosis remain rare [28]. Moreover, in this study, the authors did not perform an early detection evaluation.

Our study is directly related to the Conference and Labs for the Evaluation Forum workshop on early risk prediction on the internet (eRisk) 2017 [37], during which the authors proposed a task on the early detection of depression with a time-aware methodology and using effectiveness metrics. In general, participants based their approaches on lexical, linguistic, semantic, or statistical features, among others. We followed the workshop methodology [13,37] and used the best performing methods as baselines [38-39]. Trotzek et al [38] based their model on linguistic metainformation extracted from the subjects’ writings and developed a classifier using recurrent neural networks, whereas Villegas et al [39] explicitly modeled partial information from the semantic representation of documents using learning algorithms such as random forest (RF) or naive Bayes. Our study follows the same evaluation methodology as these studies, but it diverges from them in being a dual-model proposal, as well as in terms of the specific WFs analyzed.

## Methods

### Data Analysis

Our input comprised a set of posts and comments from a social network, specifically gathered for eRisk 2017 [13]. Data were extracted from Reddit, Inc using the Reddit, Inc’s application program interface (API), and the resulting dataset consists of a collection of tuples of the form (id, writing), such that *id* is a unique identifier for each social network user and *writing* represents a writing instance in the social network. At the same time, each writing was described as a tuple of the form (title, date, info, and text), whereby *title* indicates the title of the post or comment, *date* denotes the date and time when the writing was performed, *info* identifies the social network (in this case, only Reddit, Inc is considered), and *text* comprises the actual post or comment provided by the user. The *title* value of a comment is empty, as, in this case, the user is replying to a previous post (whose title is already defined).

Depressed users are identified by searching in the depression subreddit for posts with specific self-reports of diagnosed depression. These reports must include a more or less specific date of diagnosis. However, the errors committed in these dates are not going to interfere with the experiments because we aim at detecting if a user has been depressed or not, regardless of the concrete date of diagnosis. Moreover, a strict manual review was performed to verify that posts were genuine.

Then, a control group was created by randomly selecting a large set of redditors, including some individuals who were active on the depression subreddit but had no depression diagnosed [13]. It is important to remark that collaborating in the depression subreddit does not imply to be depressed. For instance, people trying to help others may participate in this subreddit.

The controls have not been checked for other diseases, and it is assumed that they are not depressed because they have not manifested their depression in their writings, the unique evidence used from Reddit, Inc. In fact, writings for control and depressed users are gathered from all the subreddits where the users had written, without paying attention to the concrete issues. Only users with at least 10 submissions have been considered.

The dataset has been formed starting from those writings where users claimed that they were depressed [13]. From there, a period of about a year has been considered for each user. The intervals can differ because the maximum amount of submissions that can be retrieved per redditor is 2000 (Reddit, Inc’s API limit).

As shown in Table 1, the dataset includes a total of 887 subjects, of which 135 have been diagnosed with depression, and encompasses more than 500,000 different posts and comments, with an average of nearly 600 posts per subject. In addition, other descriptive statistics are shown to demonstrate the differences between control and depressed users. On the basis of these data, we focused on estimating the likelihood that a particular subject could be considered depressed given his particular social network posts.

**Table 1:** Analysis of dataset statistics.

Features		Depressed	Control	Total
Subjects, n		135	752	887
Posts, n		49,557	481,837	531,394
**Number of submissions per subject**				
	Average	367.1	640.7	599.1
	Median (range)	154 (10-1832)	375 (10-2000)	321 (10-2000)
	Interquartile range	562	1039.5	1006
Average words per submission		27.3	21.9	22.4
**Period of time per subject (days)**				
	Average	586.42	625.02	619.15
	Median (range)	520.95 (0.60-2249.48)	477.12 (0.26-3067.16)	484.88 (0.26-3067.16)
	Interquartile range	786.88	753.19	756.83

#### Subject Behavior

To characterize the subject’s behavior on the dataset, we performed a detailed analysis of the main characteristics that might have an impact on the early detection of depression. We concentrated on variables that could be easily measured directly from the writings and in which we expected to capture certain differences in behavior between both types of subjects.

##### Textual Spreading

**Figure 1:**
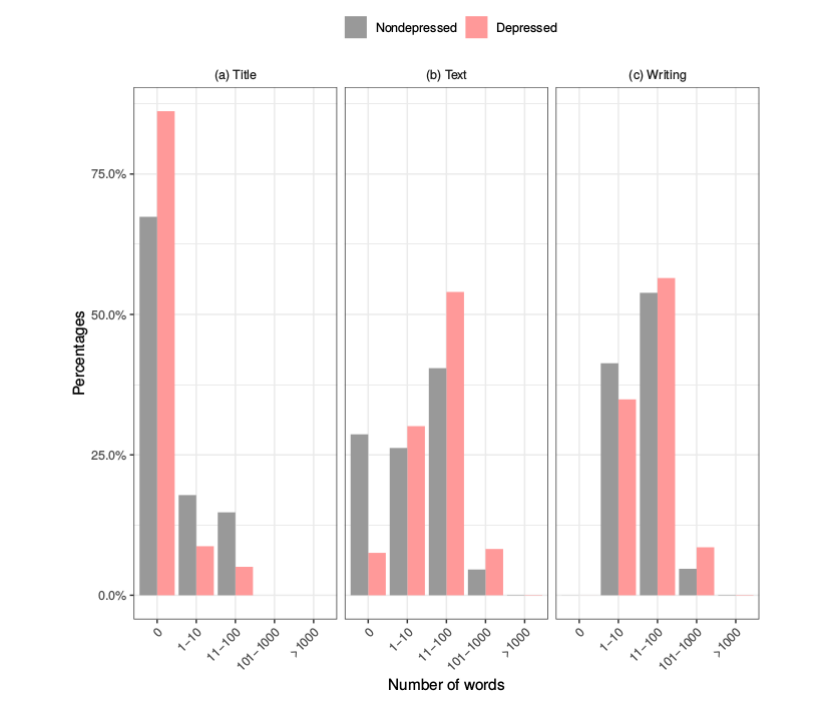
Relative percentage for number of words used on title (a), text (b), and both fields (c) for depressed and nondepressed individuals.

We began our analysis by characterizing the textual spreading of the writings produced by the subjects by measuring the number of words used in each of the writings. Figure 1 shows the words used in the post titles, both for depressed and nondepressed individuals. In particular, the number of titles with zero words (that is, comments to previous posts) is significantly higher among depressed users. That can be explained by considering how Reddit, Inc users can publish new writings: they can either publish a new reddit, for which it is mandatory to add a title; or they can comment on an already existing reddit. Thus, these results led us to conclude that depressed users have a higher tendency to reply to existing issues rather than publish new ones.

Conversely, analyzing the second plot in Figure 1, we can observe how the nondepressed users tended to send many more writings with zero words in their content description, whereas depressed subjects tended to elaborate more on their writings. In fact, the percentage of posts using between 11 and 100 words is nearly 14 points higher for depressed subjects, and it nearly doubles the percentage for even larger posts (more than 100 words). To better understand this analysis, it is important to note that there are 2 kinds of new submissions in Reddit, Inc: text submissions, whereby a user can add a text description to his title; and link submissions, in which text descriptions cannot be added, thus producing zero words in the text field.

The third plot in Figure 1 demonstrates that the total textual spreading of writings is similar for both depressed and nondepressed subjects. Although there are clear differences between the ways that depressed and nondepressed users submitted their writings, the differences in the titles are compensated for by the differences in the text, which results in similar distributions taking into account the total number of words. In any case, it is noticeable that the depressed individuals tended to elaborate their writings more and use more overall words than those who were not depressed.

These results have been checked by conducting different hypothesis contrasts. First, we employed 3 *F* tests studying the equality of variances for the number of words in title, text, and writing, considering control and depressed users. The results indicate that variances are different for title (*P*<.001) and text (*P*<.001) but equal considering the whole writing (*P*=0.62). Regarding the means, the Student *t* test computation resulted in accepting the alternative hypothesis, so the means are not equal. The *P* value is <.001 for these 3 contrasts.

##### Time Gap

**Figure 2:**
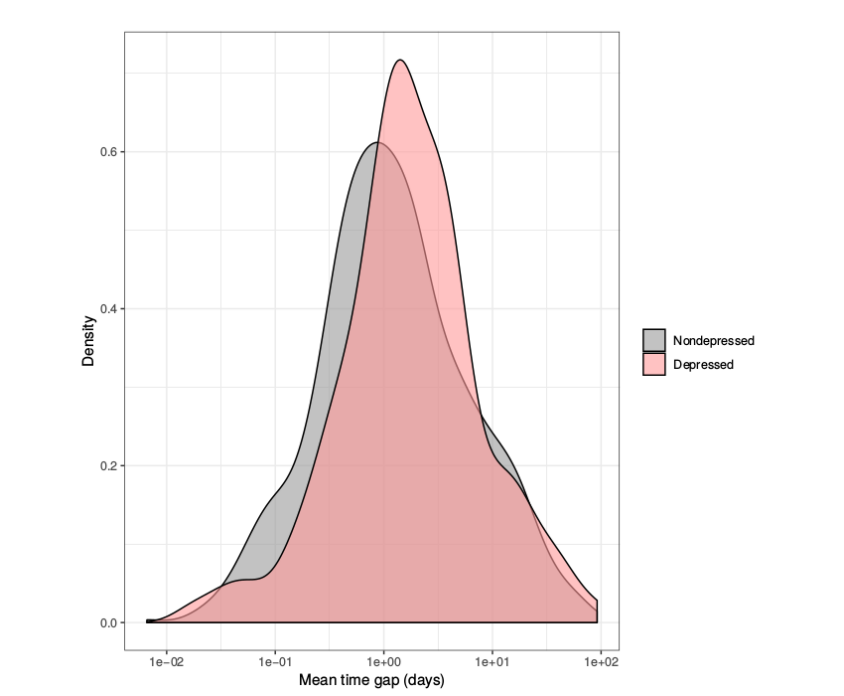
Average time gaps distribution between writings for depressed and nondepressed subjects.

Next, we focused on the time gap between 2 consecutive writings. Figure 2 displays a density plot for the time gap between writings for depressed and nondepressed individuals. In Figure 1, we can observe a higher mean among the depressed subjects, taking more time between 2 consecutive writings. In fact, the average time spent for a depressed subject between 2 writings is 5 days (5.076), whereas nondepressed writers will post again 1 day faster (4.037). In addition, the differences in the SD are significant, which is about 8 days (8.330) for nondepressed subjects but rises to 11 days (11.048) for depressed subjects. This result suggests that depressed subjects exhibit higher variability in their publication routine on the social network.

Starting from the logarithmic values of the time gap, the equality of variances was tested using an *F* test contrast. The resultant *P* value was .52, so variances are equal. In addition, the means were tested for equality between both subject types using 2-sided *t* test with significance level alpha=.05, showing that means are different (*P*=.02), which confirmed significant differences between these values among depressed and nondepressed subjects.

##### Time Span

**Figure 3:**
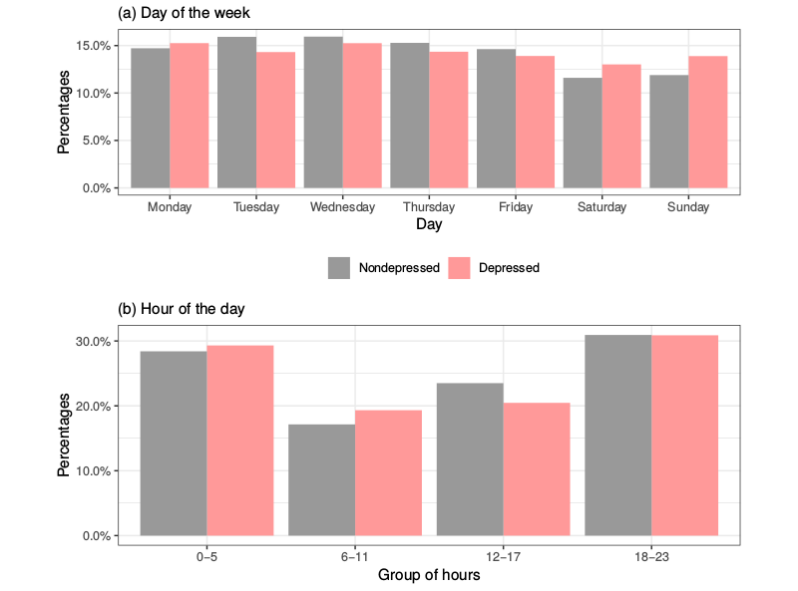
Time span bar plots according to the day of the week (a) and hour of the day (b) for depressed and nondepressed subjects.

We also explored the time span of the different writings in terms of the days and times of day when they were produced. The classification of writings according to the day of the week is described in the first plot in Figure 3. The main difference between both types is that nondepressed subjects tended to publish less during weekends than depressed individuals, whereas this tendency was inverted during weekdays, except on Mondays. In general terms, the publication rate is more homogeneous for depressed individuals, despite a small reduction at weekends. The nondepressed subjects exhibited a publication peek on Wednesdays, followed by a gradual reduction that reaches its lowest point during the weekend.

Finally, the second plot in Figure 3 shows how depressed subjects tended to send more posts and comments than nondepressed users over the hours from midnight to midday, whereas the latter published more in the afternoon. The main differences appear 6 hours before midday, when depressed subjects were most active, and 6 hours after, when nondepressed subjects were most active. The same behavior was observed by Choudhury et al [15], arguing that online activity at night is a known characteristic of these individuals, which may be the reason behind this increase.

##### Depression Prediction

The depression prediction problem presented in this study can be formalized as a binary classification problem using the presence or absence of depression diagnosis as a label. Accordingly, to address this machine learning problem, we resorted to a features-based approach and designed a collection of features that are expected to capture correlations between different aspects of the individual’s writings and depression. We represented each training example by a feature vector: φ (id, writing) ∈ R^F^, where *F* denotes the number of features, and then, we used this vector as input for the prediction function *V*. Using this approach enabled us to develop a large number of features, and we employed techniques suited for learning on a large scale, such as tree-based algorithms, to estimate relationships between those features and depression. We proposed 3 types of features: textual similarity, semantic similarity, and WFs.

#### Textual Similarity Features

Positive subjects refer to those diagnosed with MDD and vice versa for negative subjects. The main goal of these features is to estimate the degree of alignment of a subject’s writings with those of positive or negative subjects, which enables the researcher to estimate the similarity between a given subject versus positive and negative subjects. We ignored word ordering and opted for a bag-of-words representation that considered 2 different measures extensively used in the literature: cosine similarity (an instantiation of a vector space model [VSM]) and Okapi Best Matching 25 (BM25, an instantiation of a probabilistic model). The former calculates the angle formed by 2 term-frequency vectors, whereas the latter tries to estimate the probability of relevance between a query and a document.

Each subject was represented as a document that included all his writings and was modeled as a collection of words: *d*={*w*_1_, …,*w*_l_(*d*)}, where *l*(*d*) represents the number of terms in the text.

**Figure 4:**
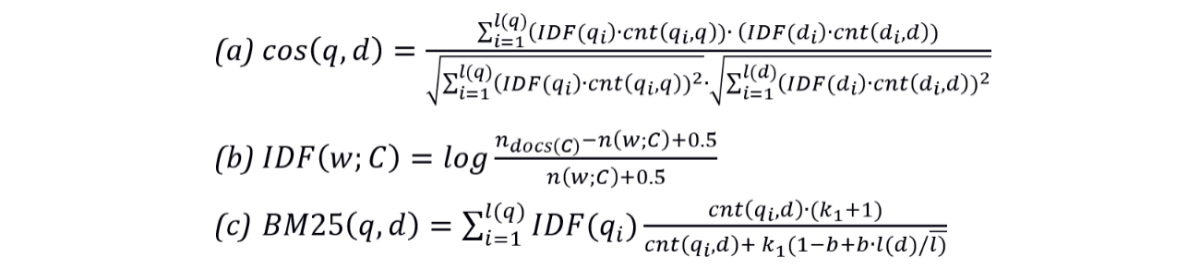
Textual similarity measures. IDF: inverse document frequency; BM25: Okapi Best Matching 25.

The cosine similarity between 2 subjects *q* and *d* is calculated as in equation a in Figure 4 following the study by Singhal [40], where *cnt* (*q*_i_, *q*) is the number of times that the term *q*_i_ appears in the document *q* and IDF(*q*_i_) is the inverse document frequency for term *q*_i_ that is computed over a corpus *C* as specified in equation b in Figure 4. In this equation, *n_docs s (C*) represents the overall number of documents in *C* (equivalent to the number of subjects), whereas *n* (*w*; *C*) is the number of documents that contain the term *w*.

The Okapi BM25 similarity between 2 subjects *q* and *d* was scored as equation c in Figure 4, following the study by Robertson and Zaragoza [41], where *k*_1_ is a scaling factor for the term frequency, *b* is a scale factor on the document length, and *l* is the average number of terms in a document. In our setting, each subject was represented by the concatenation of all the textual information available for each writing (title, info, and text), and the inverse document frequency dictionary was computed over the overall collection of these documents. The textual similarity between two subjects might have different degrees of importance, and to address this effect, we also used the aggregation of cosine and BM25 scores, with each computed between the different parts of the textual information available.

We aggregated the scores obtained for each individual’s writings by calculating the average value, SD, minimum, maximum, and median. This process was repeated for both positive and negative samples and, in all cases, the active subject was removed from the samples.

#### Semantic Similarity Features

We applied latent semantic analysis (LSA) as one of the best known VSMs to capture semantic relationships among documents. LSA explicitly learns semantic word vectors by applying singular value decomposition, which in turn projects the input word representation into a lower-dimensional space of dimensionality *k* << *V*, where semantically related words are closer than unrelated words.

In LSA, a document-term matrix *M* was constructed from a given text base of *n* documents containing *m* terms. This matrix of size *m* x *n* was then decomposed via a singular value decomposition into 3 matrices: the term vector matrix *T*; the document vector matrix *D*, and the diagonal matrix *S*:

*M=TSD*^T^ (1)

**Figure 5:**
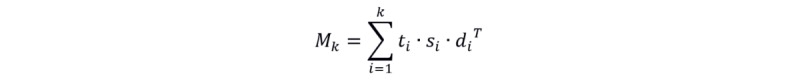
Latent semantic space.

These matrices were then reduced to the given number of dimensions *k* to result into truncated matrices *T*_k_, *S*_k_, and *D*_k_*,* creating the latent semantic space [42], as specified in Figure 5.

Different dimensionality methods have been tested in the literature. To compute the *k* dimensionality, this study typically used the Kaiser criterion [43], which will take values higher than 1.0 and return the number of singular values accordingly. We also tested the share dimensionality, which finds the first position in descending order of the singular values where their proportional sum meets or exceeds a specific share, and the fraction dimensionality, which takes a specific fraction of the number of singular values [44]; however, no relevant differences were identified among the different methods.

Semantic similarity features between 2 subjects were computed as the Euclidean distance between the respective projections into the embedding space. As described in previous section (Textual Similarity Features) , each subject was represented as a document that aggregated all of his writings. In this case, all the available textual information was used to compute the singular values. LSA was applied both following a full-text approach and removing stop words and using Porter stemming [45]. Finally, we applied feature scaling to normalize the LSA scores computed following minimum-to-maximum normalization [46]:

x’ = (x – min(x)) / (max(x) – min(x)) (2)

In this equation, *x* is the original value and *x’* is the normalized value.

#### Writing Features

The collection of features was used to profile the characteristics of the subjects’ writings on the basis of the findings from Data Analysis. As reviewed above, we defined 3 signals: textual spreading, time gap, and time span. Textual spreading measures the amount of textual information provided by the subject in his writings, and to address this feature, we introduced the following features:

- NWritings: The number of writings produced by the subject.
- AvgWords: The average number of words per writing. For each writing all the textual information available is considered.
- DevWords: SD for the number of words per writing.
- MinWords: Minimum number of words in the subject’s writings.
- MaxWords: Maximum number of words in the subject’s writings.
- MedWords: Median for the number of words in the subject’s writing.

To measure the time elapsed between 2 consecutive writings, we aggregated the writings’ time gap information for each subject. In this way, if a subject only had one writing in the time period considered, the time gap would be zero. Otherwise, the time gap would measure the number of seconds between 2 consecutive writings. A logarithmic transformation of the raw time gap values was also considered, resulting in the following 2 sets of features:

- TimeGap: The aggregated information for the time lapse between 2 consecutive writings. These values are represented as the average, SD, minimum, maximum, and median.
- LogTimeGap: For the logarithmic transformation of the time gap values. The same aggregation values are computed for each subject.

Another group of features was used to profile the moment when the writings were created by the subject. This information was expected to model differences in behavior among subjects diagnosed with depression versus those who had not been so diagnosed. The following time features were proposed:

- Day: Percentage of writings provided by the subject, for each day of the week.
- Weekday: Accumulative percentage for all writings created in a weekday.
- Weekend: Accumulative percentage for all writings posted during the weekend.
- Hour: The hours of the day are divided into 4 homogeneous classes (0:00-5:59, 6:00-11:59, 12:00-17:59, and 18:00-23:59) and the percentage of writings that fall into each class is calculated.

As a summary, textual and semantic features are computed and aggregated for each user in comparison with all other users (grouped as positive and negative), meanwhile WFs are independently calculated and aggregated for each individual with respect to his postings.

### Models

We employed a readily available machine learning toolkit [47] to develop a learning model incorporating the features that were identified. We analyzed some standard machine learning algorithms (ie, C4.5, random tree, and RF) on this classification problem and selected RF [48] as the best performing model. An independent subsampling set was used to estimate the number of trees, and BM25’s *b* and *k*_1_ metaparameters.

The evaluation followed a time-aware methodology in which the writings were chronologically sorted and grouped into subsets. Each subset was evaluated independently, and the model was required to emit 1 of 3 possible decisions:

- Depression: The subject is considered to suffer from depression. This decision is final.
- Nondepression: The subject is considered not to suffer from depression. This decision is final.
- No decision: There is not enough evidence to produce a definitive decision and it is delayed.

As this is not a traditional binary classification problem because of the delay option available when processing the different subjects’ writings, we proposed 2 different approaches: singleton and dual. The singleton model uses only 1 RF model, which is trained using the corresponding features, and a decision function is integrated to determine if enough evidence is available to proceed with a firm diagnosis or the decision must be delayed. The decision function was defined as *δ(m, th*_+_*(i), th*_-__*(i))*, where *m* denotes the machine learning model used in the binary classification problem and *th*_+_*(i)* is a threshold function that sets a limit for a positive decision depending on the information chunk being processed *(i)*, whereas *th*_-_*(i)* is a threshold function that sets a limit for a negative decision. Both threshold functions are not required to be the same, although they could be.

Different threshold functions were considered; however, the best performance was obtained with a decreasing step function. The steps of these threshold functions were tuned with a grid search over {0.95, 0.9, 0.85, 0.8, 0.75, 0.7, 0.65, 0.6, 0.55, 0.5} on the training set, and selected the best performing steps for experimentation. Finally, both threshold functions (positive and negative) are the same and follow the equation:


*th(i)* =
*0.9*_XA[1,0.9]_ +
*0.8*_XA[0.9,0.8]_ +
*0.7*_XA[0.8,0.7]_ +
*0.6*_XA[0.7,0.6]_ +
*0.5*_XA[0.6,0.5]_ (3)

In previous equation, *ΧA* (*x*) is the indicator function defined as 1 if x belongs to A, or 0 if x does not belong to A.

The main problem of the singleton approach is that it uses a binary classifier and to provide a final decision, both options (depressed or nondepressed) compete against each other and, therefore, require important support from the data features to surpass the threshold, thus causing a delay. Note that for 1 option (eg, depressed) to reach a probability of 0.9, the other option (eg, nondepressed) must be 0.1.

To overcome this matter, and inspired by the multiclass classifiers *one-versus-all* that train different binary models and select the most positive value [49], we propose the dual model that uses 2 RF models, each one trained with an independent set of features and, this way, both options do not compete but can be predicted independently. The first model (m_+_) is trained to predict depression cases, whereas the second model (m_−_) is trained to predict nondepression cases. For the dual model, a decision function of the form δ(m_+_, m_−_, th_w_, th_+_, th_−_) was defined, where m_+_ and m_−_ are the 2 learning models considered, thw denotes the number of threshold writings and th_+_ and th_−_ are the threshold functions applied to m_+_ and m_−_, respectively. Both threshold functions are defined as constant functions of the form, where the value for th_+_ is 0.9, and the value for th_−_ is 0.5.

The positive threshold function takes the upper step (0.9) from the positive threshold function of the singleton model, whereas the negative threshold function takes the lower step (0.5) of the negative threshold function of the singleton model. These thresholds were achieved following a grid search over the same values as the singleton model.

In the dual model, if the number of writings is below *th*_w_, the first model is applied with decision threshold function *th*_+_, so that if a positive probability is above the threshold, a depression decision is emitted, otherwise the decision is delayed. If the number of writings is above the writings threshold, the second model is applied with decision threshold function *th*_-_. In this case, if the nondepression probability is above the threshold the final decision is emitted and if otherwise, the decision is delayed. In this way, each classifier (*m*_+_ and *m*_-_) operates with independent features and each one can, independently, reach the threshold and provide an earlier final decision.

## Results

### Dataset

Table 2 presents the main statistics for the dataset. A total of 892 subjects were considered, of whom approximately 15% had been diagnosed with MDD. All submissions were collected from Reddit, Inc for a period covering more than 1 year [13]. Subjects with less than 10 submissions were removed.

The following evaluation is based on a subject-based train-test split, as reported in Table 2, with an approximate percentage of 55% on the training set and 45% for testing.

The sequence of writings in the test set was chronologically sorted and the set was further divided into 10 subsets (or chunks), each of which contained 10% of the messages. These subsets were considered sequentially in such a manner that the first subset contained the oldest 10% messages, the second subset the second oldest 10%, and so forth. This test subset division was a particularly important element in the evaluation, as its main objective was to detect, as soon as possible, a depression case, which would represent an improvement over traditional evaluation, which identifies cases without regard for speed. This becomes patent in the performance measure described in the next section.

### Performance Measure

Standard classification metrics such as precision, recall, or *F* measure do not take into account time, and therefore, we opted for early risk detection error (ERDE) [13]. This measure will consider both the correctness of the decision and the delay taken by the model to make the decision, where the delay is measured by the number of writings (posts or comments) seen before providing an answer.

**Figure 6:**
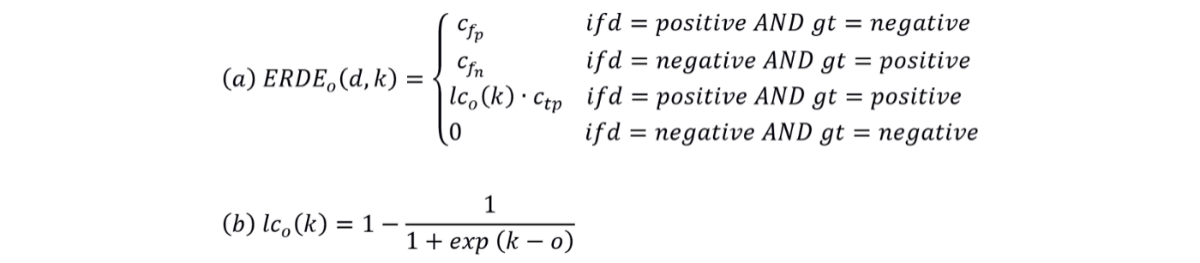
Early risk detection error metric. ERDE: early risk detection error.

Given a decision (*d*) taken by the system with a delay (*k*) and a ground truth (*gt*) for each subject, the ERDE measure is defined as equation a in Figure 6.

In that equation *c*_fp_ and *c*_fn_ are the costs associated with a false positive and false negative, respectively. In this study, following Losada and Crestani [13], *c*_fn_ was set to 1 and *c*_fn_ was set to the proportion of positive cases in the test dataset (ie, 0.1296). The correct detection of a negative does not have any repercussion (negative nor positive) in the performance of the system, independently of the moment when it is detected, as this is considered a nonrisk case that would not require an early intervention. In the case of a correct positive decision, the factor *lc*_o_(*k*) introduces a cost associated to the delay in detecting a true positive. As suggested by Losada and Crestani [13], *c*_tp_=*cf*_n_, as a late detection can have the same negative consequences as a false negative. For the *lc*_o_(*k*) factor, we use a monotonically increasing function of *k* as specified in equation b in Figure 6.

For each subject, the ERDE metric was computed, and a final score was obtained averaging all the ERDE values. As all cost weights are between 0 and 1, both included, then ERDE is also in the same range, and the quality of system performance increases as values approach 0. Following the evaluation procedure by Losada and Crestani [13], ERDE_5_ and ERDE_50_ measures were used for a comparison with the baselines, where 5 and 50 represent the subscript o for lc_o_ factor, that is, the number of writings processed from where ERDE increases more rapidly.

### Baselines

Table 3 presents the main metrics for the baselines considered. The first 3 rows contain some naïve baseline methods, the middle rows show results for some Oracle methods, and the last 2 rows expose the best performing methods from eRisk 2017 [13]. For all methods, we present the ERDE_5_ and ERDE_50_ metrics as the performance measures used in the eRisk 2017 competition, as well as *F* measure, Precision, and Recall.

Three different naïve methods that do not require any specific features (textual, semantic, or writing) were considered. The random strategy emits a random decision for each subject. As the evaluation is divided into 10 chunks, this method produces a random and equally probable verdict (*depression*, *nondepression*, or *no decision*) for each subject at the end of each chunk. As soon as the system produces a diagnosis (*depression* or *nondepression*), later decisions are not taken into account. The naïve all-depressed method will emit a *depression* decision for all subjects for all chunks. As the first chunk provides a decision for all subjects, the actions in the following chunks do not have any repercussion in the system performance. In this case, the recall reached its maximum, as expected, although both ERDE metrics obtained modest results.

**Table 2:** Dataset statistics.

Features	Training		Test	
	Depressed	Control	Depressed	Control
Subjects, n	83	403	52	349
Posts, n	30,851	264,172	18,706	217,665
Average submissions per subject	371.7	655.5	359.7	623.7
Average words per submission	27.6	21.3	26.9	22.5

**Table 3:** Baselines used for comparison with our proposed methods.

Method	ERDE^a^_5_	ERDE_50_	*F* measure	Precision	Recall
Random	18.51	15.20	0.20	0.12	0.00
All depressed	21.67	15.03	0.23	0.13	1.00
Nondepressed	12.97	12.97	0.00	0.00	0.00
Oracle1	10.38	3.74	1.00	1.00	1.00
Oracle2	11.83	5.30	1.00	1.00	1.00
Oracle3	12.23	6.73	1.00	1.00	1.00
Oracle5	12.59	7.86	1.00	1.00	1.00
Oracle10	12.97	12.97	1.00	1.00	1.00
FHDOB^b^	12.70	10.39	0.55	0.69	0.46
UNSLA^c^	13.66	9.68	0.59	0.48	0.79

^a^ERDE: early risk detection error.

^b^Model B presented by the University of Applied Sciences and Arts Dortmund, Germany (FHDO).

^c^Model A presented by the National University of San Luis, Argentina (UNSL).

We also present the nondepressed method that emits a *nondepression* decision for all subjects. As observed in Table 3, this method scored zero in all effectiveness metrics. The Oracle methods present the results for an oracle that perfectly diagnoses all subjects at the specified chunk (only results for chunks 1, 2, 3, 5, and 10 are displayed). These results prove the difficulty of this task, as the effectiveness metrics (precision, recall, and *F* measure) obtained perfect values, whereas the ERDE metrics showed error values. Oracle10 obtained the same results for nondepression because of the strict penalization of late detection of depression cases (being equivalent to a wrong diagnosis of nondepression).

Finally, the best methods from eRisk 2017 were considered for both ERDE_5_ and ERDE_50_. The FHDOB method was presented by the Biomedical Computer Science Group from the University of Applied Sciences and Arts Dortmund (Germany). This model employed linguistic metainformation extracted from the subjects’ texts and considered classifiers based on bag of words, paragraph vector, LSA, and recurrent neural networks using long short-term memory [38]. The UNSLA method was presented by the Laboratory of Research and Development in Computational Intelligence Research Group from the National University of San Luis (Argentina). This method is based on a semantic representation of documents that explicitly considers the partial information available in different chunks of data, complemented with standard categorization technology. In this case, predictions are based on their own temporal models and other sources of opinion. The LIDIC group considered multiple document representations and different learning algorithms, including RF [39].

An important difference between ERDE_5_ and ERDE_50_ is that the former promotes methods that emit few yet rapid depression decisions, whereas the latter gives smoother penalties to delays. ERDE_5_ from FHDOB and ERDE_50_ from UNSLA were used as main baselines for the comparison of our proposed methods.

### Evaluation

In this section, we present our main findings for the classification task described, and we discuss the effects of features and the performance for the different proposed models.

The first set of experiments were focused on the singleton model (Table 4).

**Table 4:** Evaluation results for the singleton model on different feature sets. Writing feature (WF) groups all WFs presented. The values for the best early risk detection error with 0=5 and 0=50 are in italics.

Features	ERDE^a^_5_	ERDE_50_	*F* measure	Precision	Recall
Cos^b^ Text^c^	15.83	13.22	0.31	0.23	0.46
Cos All^d^	16.48	13.62	0.36	0.24	0.67
BM25^e^ Text	18.11	16.61	0.26	0.16	0.60
BM25 All	14.36	12.43	0.36	0.32	0.40
LSA^f^	21.60	14.96	0.23	0.13	1.00
Norm^g^ LSA	21.34	18.02	0.23	0.13	1.00
LSA stem^h^	23.51	14.70	0.23	0.13	1.00
Norm LSA stem^i^	12.97	12.97	0.00	0.00	0.00
Cos Text + WF	14.09	13.60	0.07	0.33	0.04
Cos All + WF	13.31	12.31	0.20	0.67	0.12
BM25 Text + WF	15.59	14.62	0.29	0.24	0.37
BM25 All + WF	20.49	18.05	0.30	0.18	0.83
Cos BM25 Text + WF	14.15	12.97	0.30	0.38	0.25
Cos BM25 All + WF	13.29	12.97	0.12	0.29	0.08
LSA Cos Text + WF	17.86	12.92	0.29	0.18	0.73
LSA BM25 Text + WF	16.61	12.09	0.27	0.18	0.56
LSA Cos All + WF	19.51	13.46	0.26	0.15	0.90
LSA BM25 All + WF	20.47	14.08	0.24	0.14	0.94
LSA Cos BM25 Text + WF	18.34	12.85	0.28	0.17	0.85
LSA Cos BM25 All + WF	*12.89*	*11.27*	0.34	0.45	0.27
Norm LSA Cos Text + WF	13.35	13.35	0.04	0.20	0.02
Norm LSA BM25 Text + WF	13.58	13.33	0.11	0.17	0.08
Norm LSA Cos All + WF	14.70	14.45	0.11	0.21	0.08
Norm LSA BM25 All + WF	14.55	14.30	0.13	0.22	0.10
Norm LSA Cos BM25 Text + WF	14.60	14.60	0.25	0.25	0.08
Norm LSA Cos BM25 All + WF	13.73	13.48	0.20	0.20	0.08

^a^ERDE: early risk detection error.

^b^Cos: cosine.

^c^Only the text part of the writing is considered.

^d^The whole writing is considered.

^e^BM25: Okapi Best Matching 25.

^f^LSA: latent semantic analysis.

^g^Normalized LSA.

^h^LSA with stemming.

^i^Normalized LSA with stemming.

Initially, we tested the performance of textual and standalone LSA features, finding very low performance on the semantic features, compared with textual features, probably because of the difficulty to capture small textual details relevant to the detection of depression. The normalization of the LSA scores slightly improved the results, and the use of stemming and stop words removal had a negative impact on performance, as shown by the zero values on precision, recall, and *F* measure for normalized LSA with stemming. The best performance among textual features was obtained by BM25 using all textual writing fields (title, info, and text).

Next, we analyzed the performance of textual features combined with the WFs, as defined in Data Analysis. Curiously, BM25 performance worsened as the WFs were included, whereas cosine performance improved. However, the best results were obtained combining both textual features (cosine and BM25 similarity) with WFs using all textual writing fields.

Subsequently, the 3 feature types were combined (textual, semantic, and writings), and the best results were obtained when the textual similarity metrics (cosine and BM25) used all textual fields, altogether with LSA and WFs. The same set of experiments were executed with normalized LSA and, although the results generally improved, they did not outperform the best value for nonnormalized LSA. Focusing on the best performing singleton model from Table 4, we individually analyzed the results for the different WFs described in Data Analysis to determine these features’ behavior. Table 5 shows the results, highlighting the best ERDE_5_ and ERDE_50_ values. Best singleton model refers to *LSA Cos BM25 All* and the WFs are grouped in the following manner:

- Writing: NWritings, AvgWords, DevWords, MinWords, MaxWords, MedWords.
- TimeGap
- LogTimeGap
- Day
- Week: Weekday, Weekend
- Hour

Regarding a fast early detection (measured through ERDE_5_), the best performance was obtained just considering the text features, the time gap between writings and the publication days, which closely reflected the conclusions extracted from our data analysis on Section 3 in that a higher tendency to publish during the weekends could be observed in the depressed group. Relatively good results were also obtained combining textual features with the log time gap and writing hours (second best performance). Curiously, the combination of both week group and hour with textual features and time gap led to the worst results of the group. However, the best ERDE_5_ value from Table 4 was not outperformed by any combination, as each of these features is expected to capture different variables in the writings’ behavior.

The best value from the ERDE_50_ metric was obtained by combining text features, both time gap variants and the publication days. Three of these features obtained the best ERDE_5_ performance in Table 5. In this case, ERDE_50_ outperformed the best value from Table 4, although values are extremely close (11.26 and 11.27, respectively).

The performance values obtained in Tables 4 and 5 do not outperform our baselines, although the results are closer in the case of ERDE_5_.

Tables 6 and 7 show the performance results in terms of ERDE_5_ and ERDE_50_ for different dual model configurations. In the case of the dual model, 2 models were trained in parallel: one to detect depression cases (positive) and another to detect nondepression cases (negative). Both tables show a matrix in which the first column indicates the different features considered for the positive model, and the first row provides the features for the negative model (in the same order as the positive features).

**Table 5:** Evaluation results for classification on different writing features for the best singleton model from Table 4, which combines cosine and Okapi Best Matching 25 textual features for all text fields and latent semantic analysis features. The values for the best early risk detection error with 0=5 and 0=50 are in italics.

WF^a^ combinations	ERDE^b^_5_	ERDE_50_	*F* measure	Precision	Recall
BSM^c^ + Writing, TimeGap, Hour	17.35	11.39	0.30	0.18	0.85
BSM + Writing, TimeGap, Day	*13.59*	12.12	0.22	0.31	0.17
BSM + Writing, TimeGap, Week	14.77	11.44	0.33	0.25	0.48
BSM + Writing, LogTimeGap, Hour	14.03	13.54	0.12	0.29	0.08
BSM + Writing, LogTimeGap, Day	18.95	12.53	0.27	0.16	0.96
BSM + Writing, LogTimeGap, Week	17.80	12.72	0.28	0.17	0.85
BSM + Writing, TimeGap, Day, Hour	16.14	11.55	0.31	0.21	0.63
BSM + Writing, TimeGap, Week, Hour	19.28	12.85	0.26	0.15	0.94
BSM + Writing, LogTimeGap, Day, Hour	16.86	12.28	0.29	0.18	0.77
BSM + Writing, LogTimeGap, Week, Hour	16.91	12.13	0.29	0.19	0.63
BSM + Writing, TimeGap, LogTimeGap, Day	17.00	*11.26*	0.31	0.19	0.87
BSM + Writing, TimeGap, LogTimeGap, Week	17.85	12.62	0.30	0.18	0.87
BSM + Writing, TimeGap, LogTimeGap, Hour	17.71	12.65	0.28	0.17	0.83
BSM + Writing, TimeGap, LogTimeGap, Hour, Week	16.53	13.47	0.29	0.20	0.52

^a^WF: writing feature.

^b^ERDE: early risk detection error.

^c^BSM: best singleton model.

**Table 6:** Evaluation results for classification of different feature sets for the dual model (th_w_=6). The first column shows features for the positive model, and the first row shows features for the negative model. Positive feature sets are numbered and negative features follow the same numbering. The values for the best early risk detection error_5_ are in italics. Labels for the algorithms (Roman numerals) are shared for rows and columns.

Features	I	II	III	IV	V	VI	VII	VIII	IX	X	XI	XII
LSA^a^ (I)	13.24	12.99	12.99	12.99	12.99	12.99	13.48	13.24	13.48	13.24	29.20	13.24
Norm LSA^b^ (II)	13.22	12.97	12.97	12.97	13.47	12.97	13.47	13.22	13.47	13.22	29.43	13.22
LSA stem^c^ (III)	13.40	13.15	13.15	13.15	13.15	13.15	13.65	13.40	13.65	13.40	29.36	13.40
Norm LSA stem^d^ (IV)	13.22	12.97	12.97	12.97	13.47	12.97	13.47	13.22	13.47	13.22	29.43	13.22
Cos^e^ BM25^f^ Text^g^ + WF^h^ (V)	13.22	12.97	12.97	12.97	13.47	12.97	13.47	13.22	13.47	13.22	29.43	13.22
Cos BM25 All^i^ + WF (VI)	13.22	12.97	12.97	12.97	13.47	12.97	13.47	13.22	13.47	13.22	29.43	13.22
LSA Cos Text + WF (VII)	13.24	12.99	12.99	12.99	12.99	12.99	13.48	13.24	13.48	13.24	29.20	13.24
LSA BM25 Text + WF (VIII)	13.24	12.99	12.99	12.99	12.99	12.99	13.48	13.24	13.48	13.24	29.20	13.24
LSA Cos All + WF (IX)	12.14	11.89	11.89	11.89	11.89	11.89	12.39	12.14	12.39	12.14	28.35	12.14
LSA BM25 All + WF (X)	13.24	12.99	12.99	12.99	12.99	12.99	13.48	13.24	13.48	13.24	29.20	13.24
LSA Cos BM25 Text + WF (XI)	12.13	*11.88* ^j^	*11.88* ^j^	*11.88* ^j^	*11.88* ^j^	*11.88* ^j^	12.38	12.13	12.38	12.13	28.34	12.13
LSA Cos BM25 All + WF (XII)	12.73	12.49^j^	12.49^j^	12.49^j^	12.73	12.49^j^	12.98	12.73	12.98	12.73	28.94	12.73

^a^LSA: latent semantic analysis.

^b^Normalized LSA.

^c^LSA with stemming.

^d^Normalized LSA with stemming.

^e^Cos: cosine.

^f^BM25: Okapi Best Matching 25.

^g^Only the text part of the writing is considered.

^h^WF: writing features.

^i^The whole writing is considered.

^j^Statistically significant performance improvements over the best singleton model in Table 4.

Experiments were performed with an extensive number of feature combinations, but we have limited the results displayed on the tables to the most relevant performing features. Focusing on ERDE_5_ (Table 6), the best results were obtained when using textual features (both cosine and BM25 similarity metrics) only for the text field, LSA and WFs on the positive model, combined with LSA variants or textual features for the negative model. Among the LSA variants, except for plain LSA, normalized LSA, LSA with stemming, and normalized LSA with stemming provide the best performance. The sole use of textual similarity features (feature sets 5 and 6) with any LSA features leads to a best performing model.

We also report on statistical significance using a standard 2-sided *t* test with significance level alpha=.05 when improving performance of the best singleton model on Table 4. Significant improvements (all the *P* values obtained are smaller than 1.21e-14) over the best singleton model were obtained with positive models using both textual features (cosine and BM25) in all fields or just in the text field in combination with semantic and WFs, as well as negative models based on LSA (normalized, stemming, and both). Significant improvement was also achieved using both textual features together with WFs but skipping LSA. This suggests that all the proposed features are required to provide an early risk detection for the identification of depressed subjects, whereas a less complex model achieves better results in identifying nondepressed subjects.

Results for ERDE_50_ (Table 7) are consistent with ERDE_5_ performance (all the *P* values are smaller than .003), but the optimal value is limited to the positive model with textual features on the text field, LSA and WFs, whereas the negative model only applies LSA with stemming and removing stop words. Other best-performing models from Table 6 obtained the third best performance for ERDE_50_, whereas the second best uses cosine similarity for all text fields, LSA, and writings features for the positive model. It is remarkable that the negative model for both first and second best performance is based only on LSA with stemming and removing stop words. The dual model is able to clearly outperform the best baseline values for ERDE_5_ and ERDE_50_ from Table 3, with an improvement of 6.5% on ERDE_5_ and more than 10% improvement on ERDE_50_ over the best-performing state-of-the-art models. Thus, we were able to improve on 2 different and independent best-performing models by employing a single model with two different configurations.

**Table 7:** Evaluation results for classification of different feature sets for the dual model (th_w_=53). The first column shows features for the positive model, and the first row shows features for the negative model. Positive feature sets are numbered and negative features follow the same numbering. The values for the best early risk detection error_50_ are in italics. Labels for the algorithms (Roman numerals) are shared for rows and columns.

Features	I	II	III	IV	V	VI	VII	VIII	IX	X	XI	XII
LSA^a^ (I)	10.20	9.95	9.95	9.95	9.95	9.95	10.45	10.20	10.45	9.95	16.18	10.20
Norm LSA^b^ (II)	15.46	15.21	12.97	15.21	15.21	15.21	15.71	15.46	15.71	15.46	31.42	5.46
LSA stem^c^ (III)	11.19	10.94	10.94	10.94	10.94	10.94	11.44	11.19	11.44	11.19	25.15	11.19
Norm LSA stem^d^ (IV)	15.46	15.21	12.97	15.21	15.21	15.21	15.71	15.46	15.71	15.46	31.42	15.46
Cos^e^ BM25^f^ Text^g^ + WF^h^ (V)	15.46	15.21	12.97	15.21	15.21	15.21	15.71	15.46	15.71	15.46	31.42	15.46
Cos BM25 All^i^ + WF (VI)	15.46	15.21	12.97	15.21	15.21	15.21	15.71	15.46	15.71	15.46	31.42	15.46
LSA Cos Text + WF (VII)	10.20	9.95^j^	9.95^j^	9.95^j^	9.95^j^	9.95^j^	10.45	10.20	10.45	9.95^j^	16.18	10.20^j^
LSA BM25 Text + WF (VIII)	10.20	9.95^j^	9.95^j^	9.95^j^	9.95^j^	9.95^j^	10.45	10.20	10.45	9.95^j^	16.18	10.20^j^
LSA Cos All + WF (IX)	10.41	10.16	9.16	10.16	10.16	10.16	10.66	10.41	10.66	10.16	16.65	10.41
LSA BM25 All + WF (X)	10.32	10.07	10.07	10.07	10.07	10.07	10.57	10.32	10.57	10.07	16.30	10.32
LSA Cos BM25 Text + WF (XI)	10.17	9.93	*8.68* ^j^	9.93	9.93	9.93	10.42	10.17	10.42	9.93	17.41	10.17
LSA Cos BM25 All + WF (XII)	13.48	13.23	10.98^j^	13.23	13.23	13.23	13.73	13.48	13.73	13.48	28.94	13.48

^a^LSA: latent semantic analysis.

^b^Normalized LSA.

^c^LSA with stemming.

^d^Normalized LSA with stemming.

^e^Cos: cosine.

^f^BM25: Okapi Best Matching 25.

^g^Only the text part of the writing is considered.

^h^WF: writing features.

^i^The whole writing is considered.

^j^Statistically significant performance improvements over the best singleton model in Table 4.

## Discussion

### Principal Findings

The main findings of this study are the following: the importance of using WFs in the early detection of MDD, the comparison of the singleton and dual approaches to predict the depression condition, and the improvement of state-of-the-art algorithms, following a time-aware evaluation, obtained by the dual model.

In this paper, we presented 2 methods based on machine learning that exclusively used data from social media networks to provide an early detection of depression cases. The problem was formalized as a classification problem and was addressed using machine learning. We resorted to a features-based approach and designed a collection of features (textual, semantic, and writing) that captured correlations between different aspects of the individuals’ writings and depression. The evaluation follows a time-aware approach that rewards early detections and penalizes late detections.

Initially, we present a singleton model based on a single binary classifier and 2 threshold functions (one positive and another negative). However, the results achieved were modest because, to make a final decision, the classifier requires enough evidence to discard one option versus the other, thus causing a delay. The best results for the singleton model were obtained by combining textual and semantic similarity with all the WFs proposed. Note that an individual combination of WFs did not lead to improved results.

Our best-performing method was based on a dual approach, using a machine learning model to detect depressed subjects and another one to identify nondepressed ones. Interestingly, WFs become crucial for the positive model (in charge of detecting depression cases), along with semantic similarity and textual similarity, although limited to the post text field. On the contrary, the negative model (predicting nondepression cases) can follow a much simpler approach based on semantic or textual similarity.

In fact, focusing on ERDE_50_, the optimal value is obtained with the negative model based only on LSA with stemming and removing stop words, without considering any textual similarity or WFs. This may be related with the less strict evaluation of false negatives using this metric.

In comparison with the state-of-the-art detection models, our results showed how the dual model is able to improve performance up to more than 10%. We consider that these results can help in the development of new tools to identify at-risk individuals, enabling those people suffering from depression to be detected and receive treatment as soon as possible.

### Future Work

This study can be extended in several ways. First, we would like to extend the set of features with other document representations. Second, we plan to study different model combinations for our dual approach, with an intense focus on new machine learning algorithms and feature sets. Finally, we plan to evaluate the effectiveness of our models in different environments, such as information technologies or economics.

## Abbreviations

API: application program interface
BM25: Okapi Best Matching 25
ERDE: early risk detection error
eRisk: early risk prediction on the internet
LSA: latent semantic analysis
MDD: major depressive disorder
RF: random forest
VSM: vector space model
WF: writing feature
